# Cytochrome P450 VvCYP76F14 dominates the production of wine bouquet precursors in wine grapes

**DOI:** 10.3389/fpls.2024.1450251

**Published:** 2024-10-11

**Authors:** Guangli Xia, Matthew Shi, Weina Xu, Adeeba Dark, Zhizhong Song

**Affiliations:** ^1^ Institute of Grape Wine, College of Pharmacy, Binzhou Medical University, Yantai, Shandong, China; ^2^ Department of Plant Science, University of Cambridge, Cambridge, United Kingdom; ^3^ The Engineering Research Institute of Agriculture and Forestry, College of Horticulture, Ludong University, Yantai, Shandong, China

**Keywords:** wine grape, wine bouquet, cytochrome P450 enzyme, sequence variation, site-directed mutagenesis

## Abstract

In wine grape, the multi-functional cytochrome P450 enzyme VvCYP76F14 sequentially catalyzes the formation of linalool-derived compounds, including (*E*)-8-hydroxylinalool, (*E*)-8-oxolinalool, and (*E*)-8-carboxylinalool, which are crucial precursors for the wine bouquet. However, molecular basis towards VvCYP76F14 in regulating the wine bouquet precursor production remain unknown. In this study, both wine bouquet precursor contents and catalytic activities of VvCYP76F14s varied among the three different wine bouquet type varieties. Subcellular localization analysis revealed that VvCYP76F14s are predominantly localized in the endoplasmic reticulum. Notably, a maltose-binding protein (MBP) fusion-tag was added to each of the three VvCYP76F14 proteins in the *Escherichia coli* expression system, significantly induced the concentration of the MBP-VvCYP76F14 fusion proteins. Site-directed mutation of 4 amino acid residues (I120L, L298V, E378G, and T389A) in VvCYP76F14 resulted in a significant decrease in VvCYP76F14 enzymatic activities, respectively. Furthermore, the transient expression of *VvCYP76F14* cloned from ‘Yanniang No.2’ significantly increased the levels of (*E*)-8-hydroxylinalool, 8-oxolinalool, and (*E*)-8-carboxylinalool compounds in the transformed ‘Yanniang No.2’, ‘Italian Riesling’, and ‘Marselan’ berries, respectively. In conclusion, VvCYP76F14 dominates the production of wine bouquet precursors and could be a fingerprint marker for screening superior hybrid offspring with desired levels of wine bouquet precursors.

## 1 Introduction

In wine grape (*Vitis vinifera* L.), aroma serves as a pivotal sensory indicator of wine quality, captivating enthusiasts and connoisseurs alike (Thomas-Danguin et al., 2011; Robinson et al., 2014; Alegre et al., 2020; Riffle et al., 2022; Zhai et al., 2023). The aroma of wine principally composed of primary aromas and wine bouquet (or secondary aromas). In particular, flavor compounds within the berries contribute to primary aromas, embodied in herbal, sweet, floral, and fruity notes, while wine bouquet is formed during fermentation and ageing through biochemical processes, transforming flavor precursors into a diverse array of aromatic compounds (Lukic et al., 2017; Wang et al., 2017; Alegre et al., 2020; Riffle et al., 2022; Zhai et al., 2023). As wine ages, the primary aromas derived from the wine grape tend to diminish, while the wine bouquet becomes more prominent, destroying the typical characteristics associated with the wine’s aroma. The evolving bouquet is implicated in dominating the unique aromatic profile as the wine matures and develops (Thomas-Danguin et al., 2011; Parker et al., 2017). Notably, Picard et al. (2015) revealed a limited correlation between wine bouquet and the berry aroma directly derived from wine grape berries.

To date, a plethora of compounds contributing to the complex and diverse wine bouquet have been identified (Ghaste et al., 2015; Lin et al., 2019; Alegre et al., 2020). However, only a relatively limited amounts of compounds have been unveiled in shaping the characteristical wine bouquet (Alegre et al., 2020). Particularly, bicyclic monoterpene lactones play pivotal roles in controlling wine bouquet formation. Notably, these bicyclic monoterpene lactones are derived from the crucial precursor (*E*)-8-carboxylinalool during wine ageing (Giaccio et al., 2011; Ilc et al., 2017). The biosynthesis of (*E*)-8-carboxylinalool involves multiple steps (hydroxylation, dehydrogenation, and carboxylation), which was catalyzed by a cytochrome P450 enzyme VvCYP76F14 (Ilc et al., 2017; Lin et al., 2019; Peng et al., 2024). Unlike most of the monofunctional P450s that only catalyze a single substrate in plants, the CYP76 family enzymes are multifunctional monooxygenases capable of catalyzing multiple substrates (Paine et al., 2005). In *Arabidopsis thaliana*, the CYP76 family enzyme AtCYP76C1 can catalyze the conversion of linalool into 8-hydroxylinalool, 8-oxolinalool, and 8-carboxyllinalool as well as lilac aldehydes and lilac alcohols (Boachon et al., 2015; Kunert et al., 2023). However, the molecular basis underlying the regulation of wine bouquet precursor production by VvCYP76F14 in wine grapes are largely unknown.

In accordance with previous studies, grape varieties are typically described as ‘Neutral’ (low bouquet density), ‘Aromatic’ (middle bouquet density), and ‘Full-Bodied’ (high bouquet density), contributing differently to the wine bouquet (Giaccio et al., 2011; Thomas-Danguin et al., 2011; Yang et al., 2019; Alegre et al., 2020; Peng et al., 2024). In this study, we selected three distinct wine bouquet types (‘Italian Riesling’, Neutral; ‘Marselan’, Aromatic; ‘Yanniang No.2’, Full-bodied) to investigate sequence differences and enzymatic activities among VvCYP76F14s derived from these varieties. Subsequently, site-directed mutagenesis and *in vitro* and *in vivo* functional characterization were conducted to identify the key amino acid residues of VvCYP76F14s responsible for the three catalytic reaction processes. This study provides molecular insights for investigating the physiological function of VvCYP76F14 in wine grape and revealing the application of VvCYP76F14 as a fingerprint marker for selecting wine grape varieties with desired amounts of wine bouquet precursors.

## 2 Materials and methods

### 2.1 Chemicals

According to the description of Peng et al. (2024), (*E*)-8-hydroxylinalool, (*E*)-8-oxolinalool, and (*E*)-8-carboxylinalool were synthesized and purified in Accela ChemBio Co. Ltd. (Shanghai, China). The primary precursor of linalool was purchased from J and K Scientific Co. Ltd. (Shanghai, China). The other chemicals used in this study were purchased from China National Pharmaceutical Group Chemical Reagents Shanghai Co., Ltd (Shanghai, China).

### 2.2 Wine grape varieties and grape wine samples

*V. vinifera* cv. Italian Riesling, *V. vinifera* cv. Marselan, and *V. vinifera* × *V. labrusca* cv. Yanniang No.2 berries were collected from the National Grape Germplasm Repository in Yantai, China at 90, 100, and 110 DAFB (day after full bloom), respectively, exhibiting similar maturity stages as outlined in the Grape Grower’s Handbook (Goldammer, 2018). Berry samples were promptly frozen in liquid nitrogen for subsequent analysis. Three biological replicates were carried out, each with 40 individual wine grapes.

### 2.3 Determination of linalool-derived compounds in berries

The levels of linalool, (*E*)-8-hydroxylinalool, (*E*)-8-oxolinalool, and (*E*)-8-carboxylinalool in the wine grape berries were assessed using High-Performance Liquid Chromatography combined with High-Resolution Mass Spectrometry (HPLC-HRMS) (Waters, Milford, MA, USA) by Shanghai Bioprofile Technology Co. Ltd. (Shanghai, China).

### 2.4 Cloning and sequence analysis of *VvCYP76F14*

The coding sequences (CDSs) of *VvCYP76F14* were cloned and sequenced from three wine bouquet type varieties (‘Italian Riesling’, ‘Marselan’, and ‘Yanniang No.2’). Total RNA was extracted using the MiniBEST Plant RNA Extraction Kit (TaKaRa, Dalian, China), and the remaining DNA contamination was removed with RNase-free Recombinant DNase I (TaKaRa, Dalian, China). The quantity and quality of the extracted RNA were determined by Invitrogen Qubit Flex Fluorometer (Thermo Fisher Scientific, Waltham, USA). Then, the first-strand cDNA was synthesized using the PrimeScript II First Strand cDNA Synthesis Kit (TaKaRa, Dalian, China).

The CDSs of *VvCYP76F14* were cloned using the Prime STAR™ HS DNA polymerase (TaKaRa, Dalian, China). Specific primers of *VvCYP76F14* (Forward: 5’-ATGGAGTTGTTGAGTTGTCTG-3’; Reverse: 5’-TCAAACCCGTACAGGTAGAGCTTGCAG-3’) were synthesized in Shenggong Bioengineering Co., Ltd. (Shanghai, China). The PCR fragments were then cloned into pMD 18-T (TaKaRa, Dalian, China) and sequenced by Shenggong Bioengineering Co., Ltd. (Shanghai, China).

### 2.5 Real-time quantitative PCR

The qPCR was carried out using the LightCycler^®^ 480 system (Roche, Inc., Basel, Switzerland) and SYBR Green qPCR Master Mix (TaKaRa, Dalian, China). The positive recombinant *VvCYP76F14*-PMD 18-T plasmids were extracted using the Plasmid Miniprep Kit (Tiangen, Beijing, China), followed by the manufacturer’s instructions. The concentration of the extracted plasmids was assessed using the Qubit Flex Fluorometer (Thermo Fisher Scientific, Waltham, USA). Absolute quantification was conducted using the specific primers (Forward: 5’-TGTTATCCAACACCATAT-3’; Reverse: 5’-TCCCAGCTTCCTCCATCACA-3’) and the first-strand cDNA template. To generate a standard curve, each recombinant plasmid was diluted and used as a template for qPCR.

### 2.6 Subcellular localization of VvCYP76F14

To investigate the subcellular localization of VvCYP76F14, the CDSs of *VvCYP76F14* from ‘Italian Riesling’, ‘Marselan’, and ‘Yanniang No.2’ were cloned into the pBWA(V)HS-ccdb-GLosgfp vector (RiORUN, Wuhan, China) using *Bsa*I and *Eco*31I restriction sites, resulting in the generation of pBWA(V)HS-CYP76F14-GLosgfp constructs. The pBWA(V)HS-sper-LK-mKATE was used as an endoplasmic reticulum marker (Mravec et al., 2009). The *Agrobacterium* GV3101 strain, containing either pBWA(V)HS-CYP76F14-GLosgfp or the marker vector, was independently infiltrated into *Nicotiana benthamiana* leaves. Two days after infiltration, selected leaves were excised for confocal observations using an LSM880 microscope (Carl Zeiss, Oberkochen, Germany). The GFP fluorescence was observed using excitation/emission wavelengths of 488/510 nm, the Chlorophyll autofluorescence was observed using excitation/emission wavelengths of 640/660 nm, while the mKATE fluorescence was observed using excitation/emission wavelengths of 561/580 nm.

### 2.7 Site-directed mutagenesis

To unveil the catalytic activities of key amino acid residues in VvCYP76F14 from ‘Yanniang No.2’, alanine-scanning method was employed to generate alanine-substituted VvCYP76F14-SMs (Song et al., 2021; Peng et al., 2024). Sequence codons were optimized and synthesized at GenScript Co. Ltd. (Nanjing, China). Each of the candidate amino acid residues (N46S, T107I, N111K, I120L, R175Q, L222V, M264I, S286N, L298V, K325T, E378G, T380A, E383D, and T386A) was substituted with a corresponding amino acid with opposite polarity.

### 2.8 Heterologous expression of VvCYP76F14 in *Escherichia coli*

To enhance production and facilitate the folding of recombinant VvCYP76F14 proteins, the pMAL-c6T vector (New England Biolabs, Beijing, China) containing a maltose-binding protein (MBP) tag was used for heterologous expression (Lebendiker and Danieli, 2011). The complete CDSs of the *VvCYP76F14*s and *VvCYP76F14-SM*s, with *AlwN* I and *Sbf* I sites added at the 5’ and 3’ ends, respectively, were synthesized by GenScript Co. Ltd. (Nanjing, China). The correctness of the pMAL-c6T-VvCYP76F14s constructs was validated through sequencing (Biomarker Co. Ltd. Beijing, China), using the same primer pairs (Forward: 5’-ATGGAGTTGTTGAGTTGTCTG-3’; Reverse: 5’-TCAAACCCGTACAGGTAGAGCTTGCAG-3’). These validated constructs were then expressed in the *Escherichia coli* BL21(DE3) strain (TaKaRa, Dalian, China). The NEBExpress^®^ MBP Fusion and Purification System (New England Biolabs, Hitchin, UK) was employed to facilitate the purification of MBP-VvCYP76F14s, using the affinity between MBP and amylose resin. Subsequently, the MBP-VvCYP76F14s were cleaved from the MBP-tag using TEV Protease (New England Biolabs, Hitchin, UK) and purified using sodium dodecyl sulfate polyacrylamide gel electrophoresis (SDS-PAGE) assay. The confirmation of the recombinant proteins was performed using HPLC-HRMS (Waters, Milford, MA, USA).

### 2.9 *In vitro* enzymatic activity assay

*In vitro* enzyme activities of recombinant VvCYP76F14 from ‘Yanniang No.2’ (Full-Bodied) and VvCYP76F14-SMs were determined using linalool, (*E*)-8-hydroxylinalool, and (*E*)-8-oxolinalool as the substrate, respectively. Preliminary assays were conducted to determine the optimum reaction system. The *Arabidopsis* NADPH cytochrome P450 reductase (ATR1, Werck-Reichhart and Feyereisen, 2000; Jensen and Møller, 2010; Lin et al., 2019; Kunert et al., 2023) was chosen as the electron transport redox partner of VvCYP76F14. The enzyme activity assays were conducted in a final volume of 5 mL, supplied with 100 mM Na^+^/K^+^ phosphate buffer (pH 5.0), 1 mM NADPH, and adjusted ratio of VvCYP76F14 to ATR1 (2:1). The reactions were conducted at 25 ± 1°C for 1 h with agitation, and the resulting product was gathered and determined using HPLC-HRMS (Waters, Milford, MA, USA). Boiled protein (nonfunctional) was taken as the control. For the determination of kinetic parameters, substrate reduction was both qualitatively and quantitatively determined. The turnover number (*k*_cat_) and affinity (*k*_m_) were individually calculated. All assays were conducted in sextuplicate.

### 2.10 Overexpression of full-bodied variety *VvCYP76F14* in neutral variety berries

To assess the enzymatic activity of VvCYP76F14 in berries, the CDS of *VvCYP76F14* was isolated from the Full-Bodied variety ‘Yanniang No.2’ and synthesized by GenScript Co. Ltd. (Nanjing, China), and further cloned into the pMDC32-HPB vector (Addgene: 32078) to generate the recombinant plasmid pMDC32-HPB-*VvCYP76F14*. Subsequently, the recombinant plasmid and empty vector were transferred into the *Agrobacterium* GV3101 strain (WEIDI, Shanghai, China), respectively. In detail, 1 mL of the *Agrobacterium* GV3101 suspension with an OD_600_ of 0.8 was injected into the berries of ‘Yanniang No.2’ at 110 days after full bloom (DAFB), ‘Marselan’ at 100 DAFB, or ‘Italian Riesling’ at 90 DAFB, in the early morning (approximately 24-26 °C) at four points individually around the diameter of the wine grape surface. After injection for 3 days, the contents of linalool, (*E*)-8-hydroxylinalool, (*E*)-8-oxolinalool, and (*E*)-8-carboxylinalool in the grape berries were determined by HPLC-HRMS (Waters, Milford, MA, USA).

### 2.11 Statistical analysis

The significant differences were conducted in IBM SPSS Statistics 23 (Armonk, New York, USA), using ANOVA followed by Fisher’s LSD test method.

## 3 Results

### 3.1 Different wine bouquet precursor contents among neutral, aromatic, and full-bodied wine grape varieties

**Figure 1:**
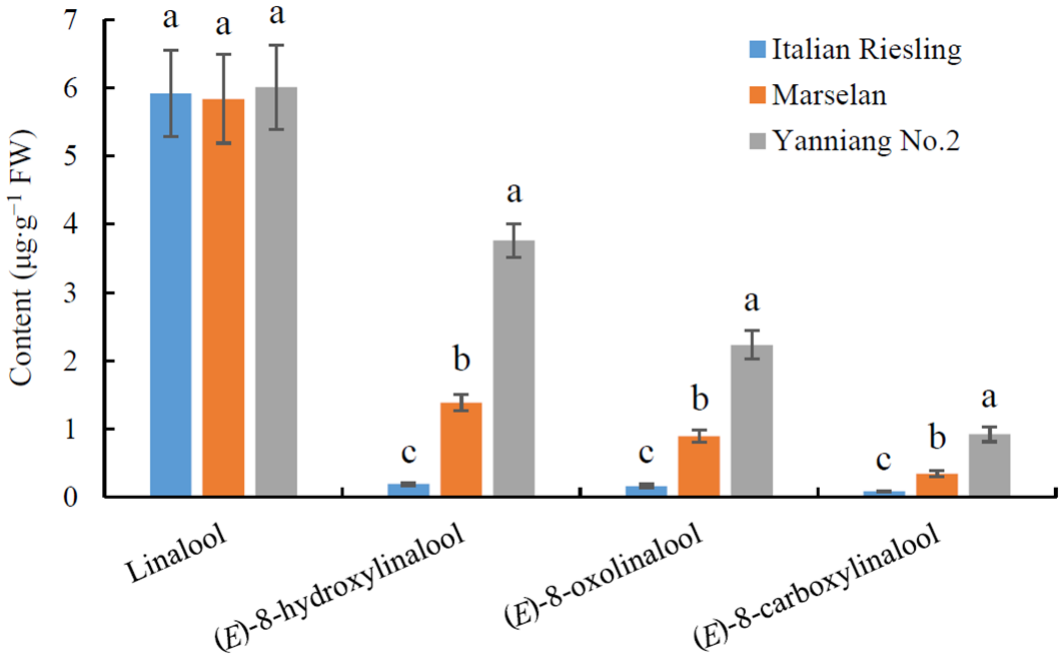
Determination of wine bouquet precursor contents from three typical wine bouquet types of grape varieties. *Vitis vinifera* cv. Italian Riesling, *V. vinifera* cv. Marselan, and *V. vinifera* × *V. labrusca* cv. Yanniang No.2 berries were collected at 90, 100, and 110 DAFB (day after full bloom), respectively. The contents of linalool, (*E*)-8-hydroxylinalool, (*E*)-8-oxolinalool and (*E*)-8-carboxylinalool in the grape berries were determined using HPLC-HRMS. Data are presented as means ± SEs (*n* = 3). Letters represent significant differences among three wine bouquet type varieties at a significance level of *p* ≤ 0.05, as determined using ANOVA followed by Fisher’s LSD test.

The core collection of the National Grape Germplasm Repository in Yantai, China has proven to be a valuable resource for studying and evaluating the contributions of various wine grape varieties to wine bouquet (Xiao et al., 2020; Peng et al., 2024). An analysis of wine bouquet precursor contents revealed that there was no difference in the levels of linalool among ‘Italian Riesling’ (Neutral), ‘Marselan’ (Aromatic), and ‘Yanniang No.2’ (Full-bodied) berries (**Figure 1**; **Supplementary Table 1**). However, the levels of (*E*)-8-hydroxylinalool, 8-oxolinalool, and (*E*)-8-carboxylinalool varied significantly among the three wine bouquet type varieties, respectively (**Figure 1**). Notably, the maximum amounts of (*E*)-8-hydroxylinalool, 8-oxolinalool, and (*E*)-8-carboxylinalool were observed in ‘Yanniang No.2’ berries, followed by Marselan’ and ‘Italian Riesling’ berries (**Figure 1**).

### 3.2 Identification of amino acid residue variations in VvCYP76F14s

**Figure 2:**
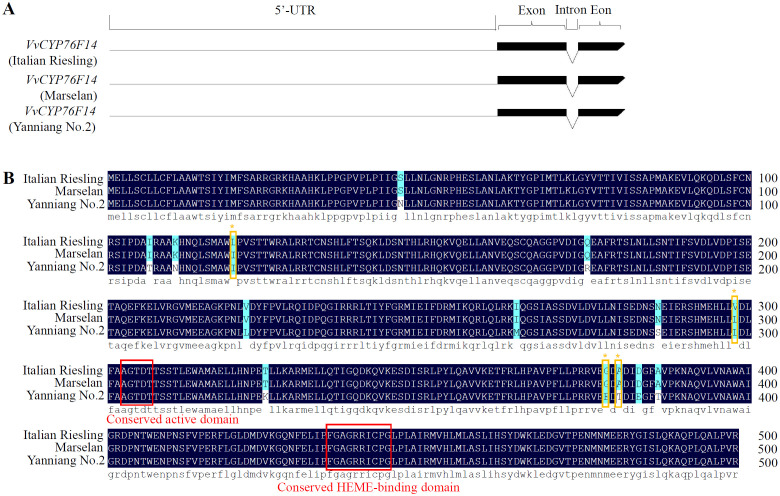
Gene structure and amino acid sequence alignment analysis. **(A)** Gene structures of *VvCYP76F14s* from three typical wine bouquet types of grape varieties. **(B)** Amino acid sequence alignment revealed amino acid residue variations in VvCYP76F14s derived from Neutral and Aromatic wine grape varieties. Multiple sequence alignment was performed on the amino acid sequences of VvCYP76F14s from wine grape varieties representing three types of wine bouquet (*V. vinifera* cv. Italian Riesling, *V. vinifera* cv. Marselan, and *V. vinifera* × *V. labrusca* cv. Yanniang No.2). Putative key amino acid residues in the Neutral variety (Italian Riesling) were indicated by brown frames and stars. Conserved regions are highlighted in red frames. The alignment analysis was conducted using the ClustalW program within the MEGA 13.0 software.

To investigate sequence differences among these three wine grape varieties, we isolated the CDSs of *VvCYP76F14*s from ‘Italian Riesling’ (Neutral), ‘Marselan’ (Aromatic), and ‘Yanniang No.2’ (Full-bodied) berries. Sequencing results revealed that all three *VvCYP76F14*s possessed the identical gene structure that contained with a long 5’-UTR region, two exons, and one intron, respectively (**Figure 2A**). In addition, all three *VvCYP76F14*s encoded a predicted polypeptide consisting of 499 amino acids (**Figure 2B**). In particular, sequence variations were observed among these three wine grape varieties. Compared to ‘Yanniang No.2’, 14 site variations (N46S, T107I, N111K, I120L, R175Q, L222V, M264I, S286N, L298V, K325T, E378G, T380A, E383D, and T386A) were found in ‘Italian Riesling’ and 12 site variations (N46S, N111K, R175Q, L222V, M264I, S286N, L298V, K325T, E378G, T380A, E383D, and T386A) were observed in ‘Marselan’ (**Figure 2B**). Furthermore, the expression levels of *VvCYP76F14* exhibited no differences in berries of these three varieties (**Supplementary Figure 1**).

### 3.3 VvCYP76F14 is localized in the endoplasmic reticulum

**Figure 3:**
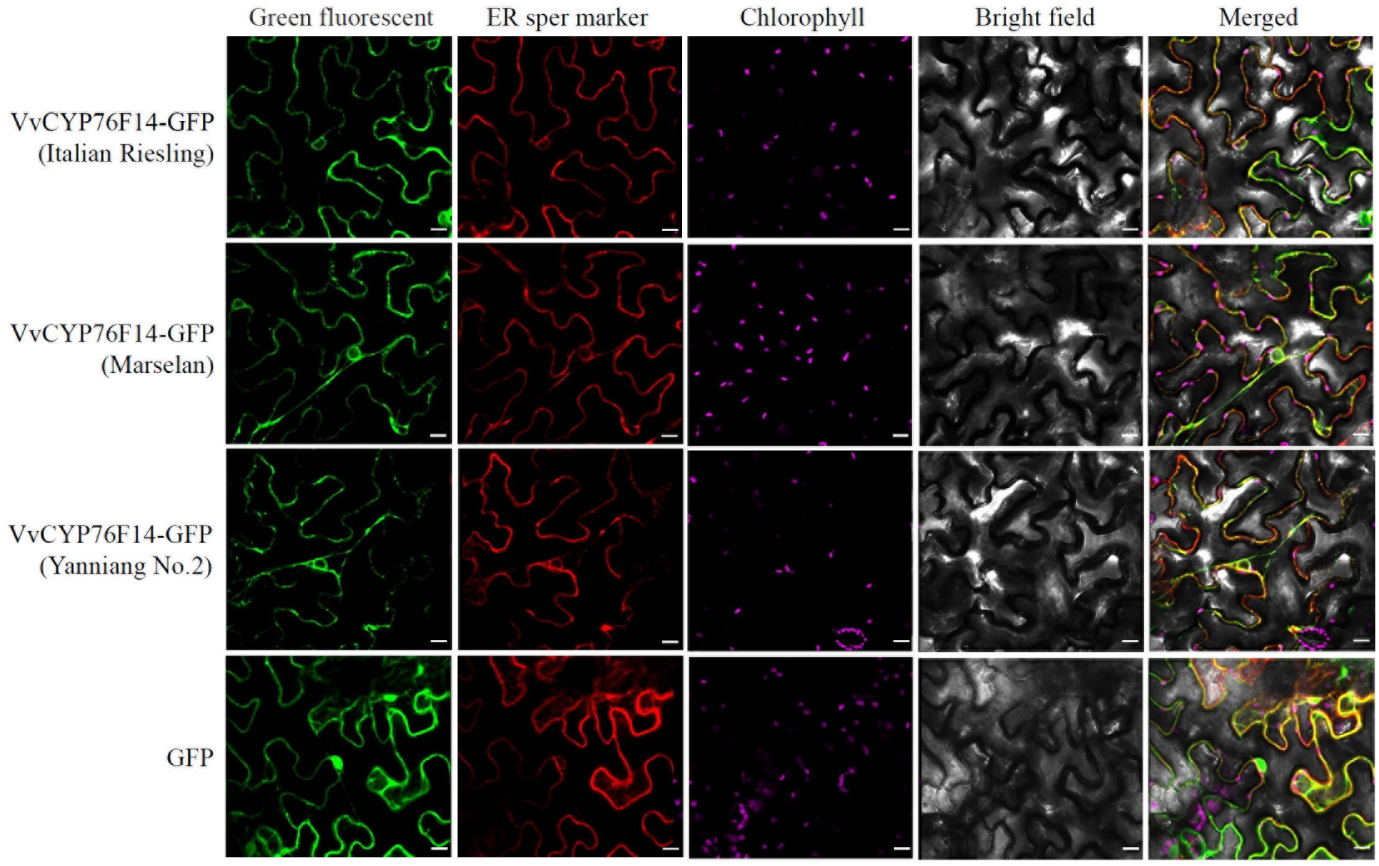
Subcellular localization analysis of VvCYP76F14s from three typical wine bouquet type varieties. The coding sequence of *VvCYP76F14s* derived from ‘Italian Riesling’ (Neutral), ‘Marselan’ (Aromatic), or ‘Yanniang No.2’ (Full-bodied) was cloned into the pBWA(V)HS-ccdb-GLosgfp vector (RiORUN, Wuhan, China). The pBWA(V)HS-sper-LK-mKATE was used as an endoplasmic reticulum marker. The *Agrobacterium* GV3101 strain containing either the pBWA(V)HS-CYP76F14-GLosgfp or the marker vector was independently infiltrated into *Nicotiana benthamiana* leaves. Two days after infiltration, selected leaves were excised for confocal observations using a LSM880 microscope. The GFP fluorescence was observed using excitation/emission wavelengths of 488/510 nm, the Chlorophyll autofluorescence was observed using excitation/emission wavelengths of 640/660 nm, while the sper mKATE fluorescence was observed using excitation/emission wavelengths of 561/580 nm. Scale bar = 10 μm.

In this study, the coding sequence of *VvCYP76F14s* derived from ‘Italian Riesling’ (Neutral), ‘Marselan’ (Aromatic), or ‘Yanniang No.2’ (Full-bodied) was cloned into the pBWA(V)HS-ccdb-GLosgfp vector (RiORUN, Wuhan, China) and expressed in *N. benthamiana* leaves, respectively. The pBWA(V)HS-sper-LK-mKATE was used as an endoplasmic reticulum marker. Confocal observations revealed that all VvCYP76F14s derived from ‘Italian Riesling’ (Neutral), ‘Marselan’ (Aromatic), and ‘Yanniang No.2’ (Full-bodied) were predominantly localized in the endoplasmic reticulum (**Figure 3**).

### 3.4 Full-bodied type VvCYP76F14 exhibited higher enzymatic activity *in vitro*

**Figure 4:**
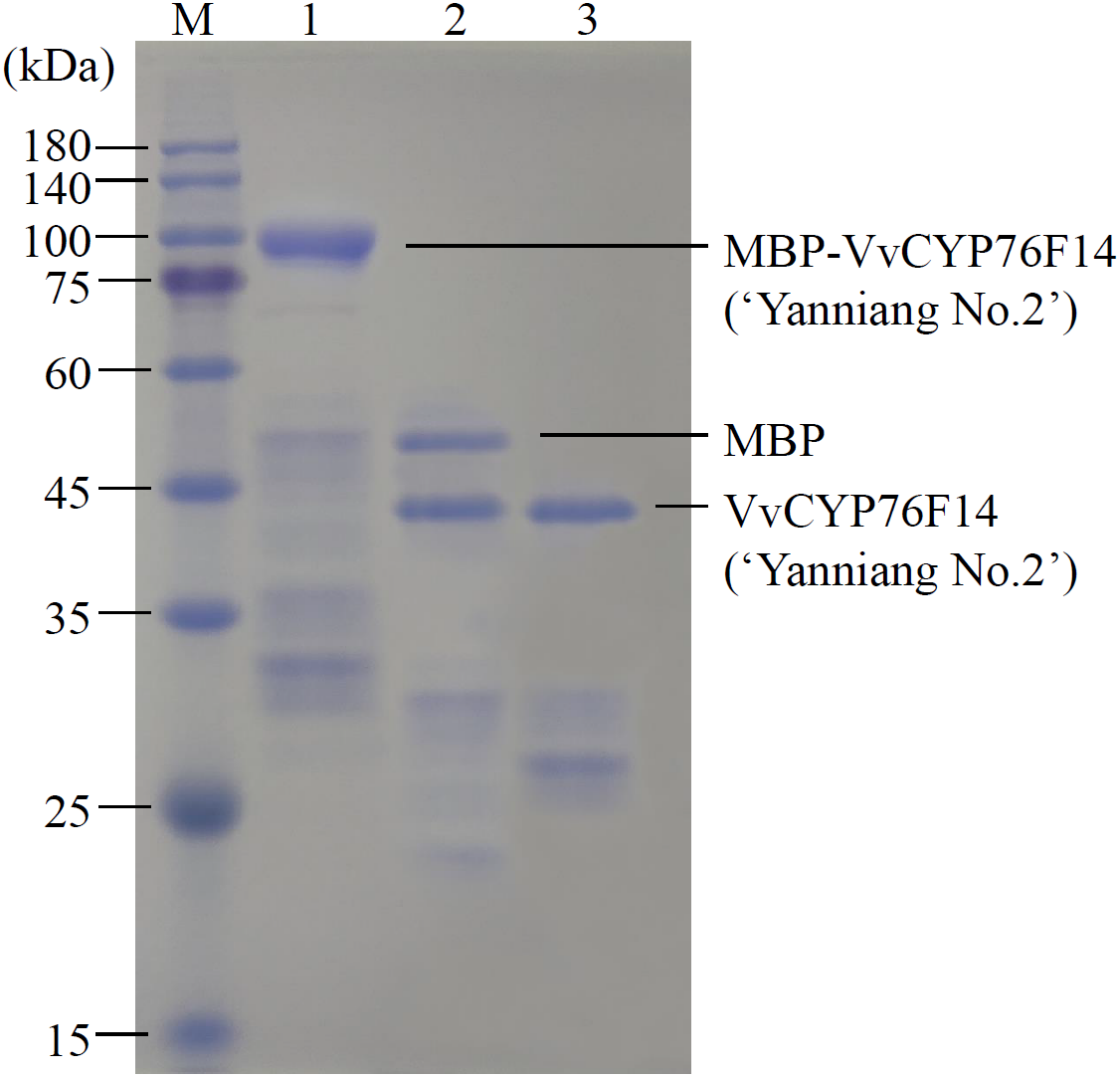
SDS-PAGE of MBP fusion analysis of VvCYP76F14s. SDS-PAGE analysis of the recombinant VvCYP76F14 from the Full-bodied variety of ‘Yanniang No.2’ in *E. coli*. The pMAL-c6T-VvCYP76F14s (Biomarker Co., Ltd, Beijing, China) constructs were expressed in the *E. coli* BL21(DE3) strain. The protein standard marker (M) was loaded on the gel. Lane 1 represents purified MBP-VvCYP76F14 eluted from amylose column with maltose. Lane 2 represents purified VvCYP76F14 after TEV protease cleavage. Lane 3 represents isolated and partially purified VvCYP76F14.

Considering the previously observed low concentration of VvCYP76F14 produced (too low to quantify) in a heterologous expression system (Boachon et al., 2015; Ilc et al., 2017), a maltose-binding protein (MBP) fusion-tag was added to each of the three VvCYP76F14 proteins in the *E. coli* expression system. Taking the Full-Bodied type VvCYP76F14 (‘Yanniang No.2’) for example, this modification resulted in a significantly higher concentration of the MBP-VvCYP76F14 fusion protein (**Figure 4**). Subsequently, the MBP-VvCYP76F fusion was cleaved using TEV protease, resulting in the isolation of VvCYP76F (**Figure 4**).

**Figure 5:**
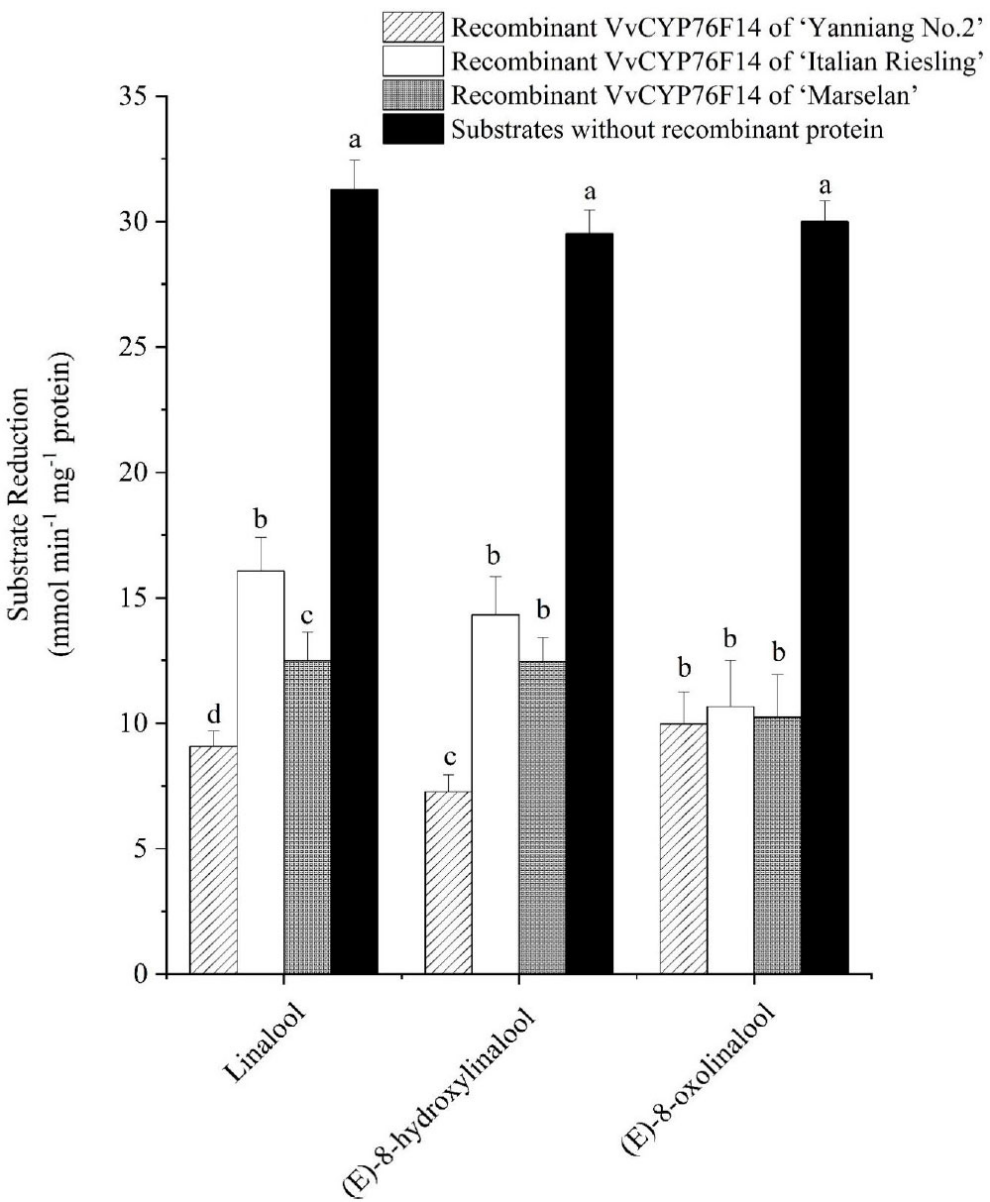
The *in vitro* specific activity of the recombinant VvCYP76F14s from *E*. *coli*. The *in vitro* specific activity of three wine bouquet type VvCYP76F14s was assessed by measuring the residual levels of substrates. Data are presented as means ± SE (*n* =3). Letters indicate significant differences among VvCYP76F14s from three wine bouquet type varieties at a significance level of *p* ≤ 0.05, as determined using ANOVA followed by Fisher’s LSD test.

The subsequent enzyme activity analysis involved monitoring the decrease in the substrate concentration. All three VvCYP76F14s demonstrated catalytic activity in reactions utilizing linalool (hydroxylation), (*E*)-8-hydroxylinalool (dehydrogenation), and (*E*)-8-oxolinalool (carboxylation) as substrates (**Figure 5**; **Supplementary Table 2**). In detail, the Full-Bodied type VvCYP76F14 exhibited a significantly higher depletion of linalool and (*E*)-8-hydroxylinalool substrates, compared to the other two types, reflects the fact that the catalytic activity of ‘Yanniang No.2’ VvCYP76F14 was higher than those of ‘Italian Riesling’ and ‘Marselan’ VvCYP76F14s. However, there were no noticeable differences in catalytic efficiencies among the three VvCYP76F14s when using (*E*)-8-oxolinalool as a substrate, means that no significant difference in the catalytic activity of three VvCYP76F14s. Furthermore, there were no significant changes in the catalytic activities utilizing linalool, (*E*)-8-hydroxylinalool, or (*E*)-8-oxolinalool as substrate, respectively, for the empty controls without recombinant VvCYP76F14s (**Figure 5**).

### 3.5 Substitution of key amino acid residues diminishes activity of VvCYP76F14 *in vitro*

In particular, 14 site variations and 12 site variations were observed in VvCYP76F14 of ‘Italian Riesling’ and ‘Marselan’, respectively, compared to ‘Yanniang No.2’ (**Figure 2B**). To estimate the individual contribution of each candidate amino acid residue to the reactions of VvCYP76F14 from ‘Yanniang No.2’, their functions were examined using site-directed mutagenesis (SM). VvCYP76F14 and VvCYP76F14-SMs were heterologously expressed in *E. coli* as above and further identified by HPLC-HRMS.

**Figure 6:**
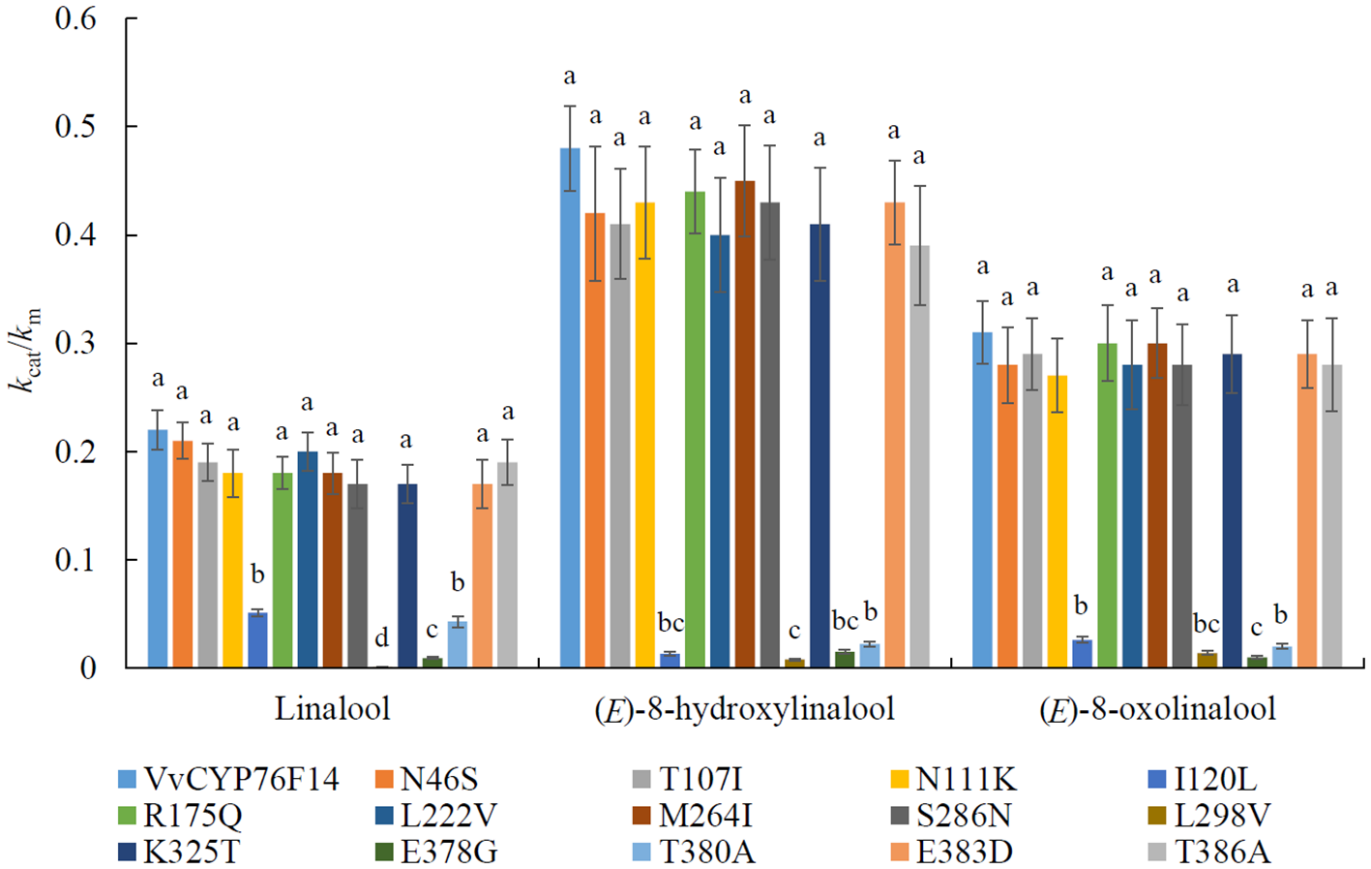
Enzyme kinetics of VvCYP76F14 and its site-directed mutant proteins (VvCYP76F14-SMs) using linalool, (*E*)-8-hydroxylinalool and (*E*)-8-oxolinalool as substrate, respectively. VvCYP76F14 was isolated from the Full-Bodied variety ‘Yanniang No.2’. The reaction product was collected and analyzed using HPLC-HRMS (Waters, Milford, MA, USA). Boiled protein (nonfunctional) served as a control. For the determination of kinetic parameters, substrate reduction was both qualitatively and quantitatively determined by HPLC-HRMS (Waters, Milford, MA, USA). The turnover number (*k*_cat_) and affinity (*k*_m_) were individually calculated. Data were presented as the means ± SE (*n* = 3). Letters represent significant differences among VvCYP76F14 and VvCYP76F14-SMs at a significance level of *p* ≤ 0.05, as determined using ANOVA followed by Fisher’s LSD test.

Based on the enzyme kinetic analyses, the substitution of I120L, L298V, E378G, and T389A lead to a significant reduction in the *k*_cat_/*k*_m_ ratios, correspondingly, implying decreased activities in the respective VvCYP76F14-SMs (**Figure 6**; **Supplementary Table 3**). However, there is no differences in enzymatic activity between the remaining 10 VvCYP76F14-SMs (N46S, T107I, N111K, R175Q, L222V, M264I, S286N, K325T, E383D, and T386A) and the wild type VvCYP76F14, respectively (**Figure 6**).

### 3.6 Transient expression of the full-bodied type *VvCYP76F14* resulted in the restoration of wine bouquet precursor levels in the each of the three wine bouquet varieties

**Figure 7:**
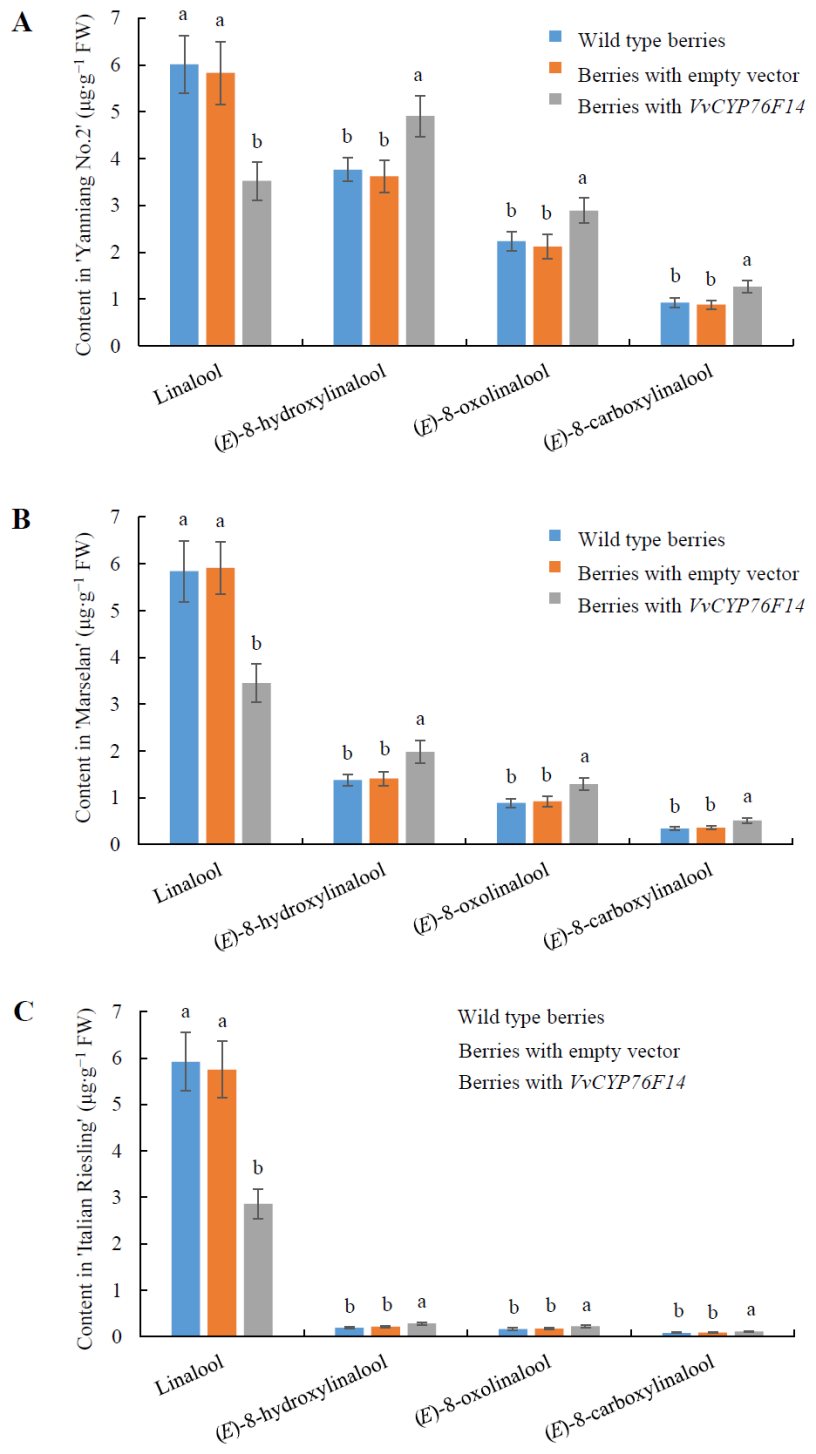
Transient expression of Full-Bodied type *VvCYP76F14* in three wine bouquet type varieties. The CDS of *VvCYP76F14* was isolated from the Full-Bodied variety ‘Yanniang No.2’ and further cloned into the pMDC32-HPB (Addgene: 32078) to generate the recombinant plasmid pMDC32-HPB-*CYP76F14*. The pMDC32-HPB-*VvCYP76F14* overexpression vector and empty vector were transferred into *Agrobacterium* GV3101 (WEIDI, Shanghai, China) strain, respectively. The *Agrobacterium* GV3101 suspension with an OD_600_ of 0.8 was injected into the berries of ‘Yanniang No.2’ at 110 DAFB **(A)**, ‘Marselan’ at 100 DAFB **(B)**, or ‘Italian Riesling’ at 90 DAFB **(C)**, respectively. After injection for 3 days, the contents of linalool, (*E*)-8-hydroxylinalool, (*E*)-8-oxolinalool and (*E*)-8-carboxylinalool in the grape berries were determined using HPLC-HRMS. Data are presented as means ± SEs (*n* = 3). Letters represent significant differences among wild type berries, berries transformed with empty vector, and berries transformed with *VvCYP76F14* at a significance level of *p* ≤ 0.05, as determined using ANOVA followed by Fisher’s LSD test. FW means fresh weight.

To further investigate the physiological function of VvCYP76F14 *in vivo*, pMDC32-HPB-*VvCYP76F14* (containing the CDS of *VvCYP76F14* isolated from the Full-Bodied variety ‘Yanniang No.2’) was introduced into ‘Yanniang No.2’, ‘Marselan’, and ‘Italian Riesling’ berries, respectively. Results showed that the levels of (*E*)-8-hydroxylinalool, 8-oxolinalool, and (*E*)-8-carboxylinalool compounds in the transformed ‘Yanniang No.2’ (**Figure 7A**), ‘Marselan’ (**Figure 7B**), and ‘Italian Riesling’ (**Figure 7C**) berries carrying pMDC32-HPB-*VvCYP76F14* were significantly higher than that of wild-type berries and berries transformed with the empty vector, whereas the levels of linalool were significantly reduced in all tested *VvCYP76F14* overexpressing berries (**Figure 7**; **Supplementary Table 4**).

## 4 Discussion

In wine grapes, the cytochrome P450 VvCYP76F14 can catalyze trisubstrate-triproduct reaction processes (hydroxylation, dehydrogenation and carboxylation) to produce (*E*)-8-carboxylinalool, which is an important wine bouquet precursor during wine making and wine ageing processes (Ilc et al., 2017; Yang et al., 2019; Zhai et al., 2023). However, physiological function of wine grape VvCYP76F14 in regulating the production of wine bouquet precursors remains unknown.

Picard et al. (2015) revealed that there is limited correlation between wine bouquet and the berry aroma derived directly from wine grape berries. In contrast, we found significant variations in catalytic activity among different wine grape varieties (mutants) of the VvCYP76F14 enzyme. Simultaneously, numerous genetic differences have also been observed in 1-deoxy-dxylulose 5-phosphate synthase (Battilana et al., 2009), terpene synthase (Martin et al., 2010), and glycosyl transferase (Schwab et al., 2014; Bönisch et al., 2014) among different varieties that have the potential to enhance wine bouquet quality (Lin et al., 2019).

*In vitro* enzymatic assays have been commonly used to characterize CYP450 family members in plants, including those from *Solanum tuberosum* (Grausem et al., 2014), *A. thaliana* (Boachon et al., 2015), and *Salvia miltiorrhiza* (Li et al., 2021). It is worthy noticing that sequence differences of VvCYP76F14 between two wine grape varieties were also identified in the study of Ilc et al. (2017), but no qualitative differences were found *in vitro* between the two VvCYP76F14 enzymes. This could be due to the limited amount of recombinase obtained for quantitative analysis and the presence of nonsense mutations in many of the identified variation sites. In this present study, we investigated the key amino acid residues of VvCYP76F14 obtained from a Full-Bodied variety and elucidated their contribution to the enzyme activity through enzymatic assays via the help of MBP fusion-tag expression system in *E. coli*. All VvCYP76F14s from three bouquet types could catalyze the monoterpenol reaction using linalool, (*E*)-8-hydroxylinalool, and (*E*)-8-oxolinalool as substrates, and the Full-Bodied type VvCYP76F14 definitely exhibited the strongest enzyme activity, which was in line with the findings of Peng et al. (2024).

To be honest, we tried and failed to obtain the crystal structure of VvCYP76F14 in this present study. According to the reported crystal structure of *S. miltiorrhiza* CYP76 homologs (Protein Data Bank no. 5YLW, Li et al., 2021), their active center is A (G) G (A) XD (E) T, which is consistent with the conserved active domain of three wine grape VvCYP76F14s (**Figure 2B**). This directly proves that mutation of key amino acid residues in VvCYP76F14s is similar to *S. miltiorrhiza* CYP76 homologs, and the functional amino acid residues are located around the active center (Li et al., 2021). Mutating key enzymes to select cultivars with desired characteristics has been well documented in various crops (Karunarathna et al., 2020; Zhang et al., 2022). Among distinct fruit crop cultivars, substitutions in key enzymes may dominant the phenotypic variability in aromatic component contents (Dunemann et al., 2012; Peng et al., 2020; Song et al., 2021). In this present study, key amino acid variations (I120L, L298V, E378G, and T389A) in VvCYP76F14 were observed in ‘Italian Riesling’ (Neutral) and ‘Marselan’ (Aromatic) berries, which might be implicated in all three reactions that significantly decreased the enzymatic activities of VvCYP76F14-SMs *in vitro*. These findings indicate that these substitutions are responsible for decreased levels of wine bouquet precursors. Furthermore, the overexpression of the Full-Bodied *VvCYP76F14* in all the three wine bouquet type varieties validated the role of VvCYP76F14 in linalool-derivative production *in vivo*, which are in line with the *in vitro* catalytic capabilities of VvCYP76F14, implying a close relationship among key amino acid substitutions, VvCYP76F14 activity, and linalool-derivative production.

Favorably, VvCYP76F14 may be a selective marker to screen VvCYP76F14 variants in grape varieties that contribute differently to wine bouquet. Nonetheless, the wine grape VvCYP76F14 is implicated in dominating the production of wine bouquet precursors.

## 5 Conclusion

The present study underscores the identification of key amino acid substitutions in VvCYP76F14s derived from different wine grape cultivars. Site-directed mutation of 4 amino acid residues (I120L, L298V, E378G, and T389A) in VvCYP76F14 resulted in a significant decrease in enzyme activities. Transient expression of *VvCYP76F14* cloned from high wine bouquet ‘Yanniang No.2’ significantly increased the levels of (*E*)-8-hydroxylinalool, 8-oxolinalool, and (*E*)-8-carboxylinalool compounds in the transformed ‘Yanniang No.2’, ‘Italian Riesling’, and ‘Marselan’ berries. This study provides an opportunity to use VvCYP76F14 as a fingerprint marker for screening hybrid offspring with desired levels of linalool-derivatives.

## Data Availability

The raw data supporting the conclusions of this article will be made available by the authors, without undue reservation.

## Supplementary material

Supplementary Figure 1 Expression levels of *VvCYP76F14s* from three wine bouquet types of grape varieties. Data are presented as means ± SEs (*n* = 3). Letters represent significant differences at a significance level of *p* ≤ 0.05, as determined using ANOVA followed by Fisher’s LSD test.
