# Supplementary figures and images for: Cytochrome P450 VvCYP76F14 dominates the production of wine bouquet precursors in wine grapes
Source: Front Plant Sci. 2024 Oct 11;15:1450251. doi: 10.3389/fpls.2024.1450251 (PMC11502375; doi:10.3389/fpls.2024.1450251)

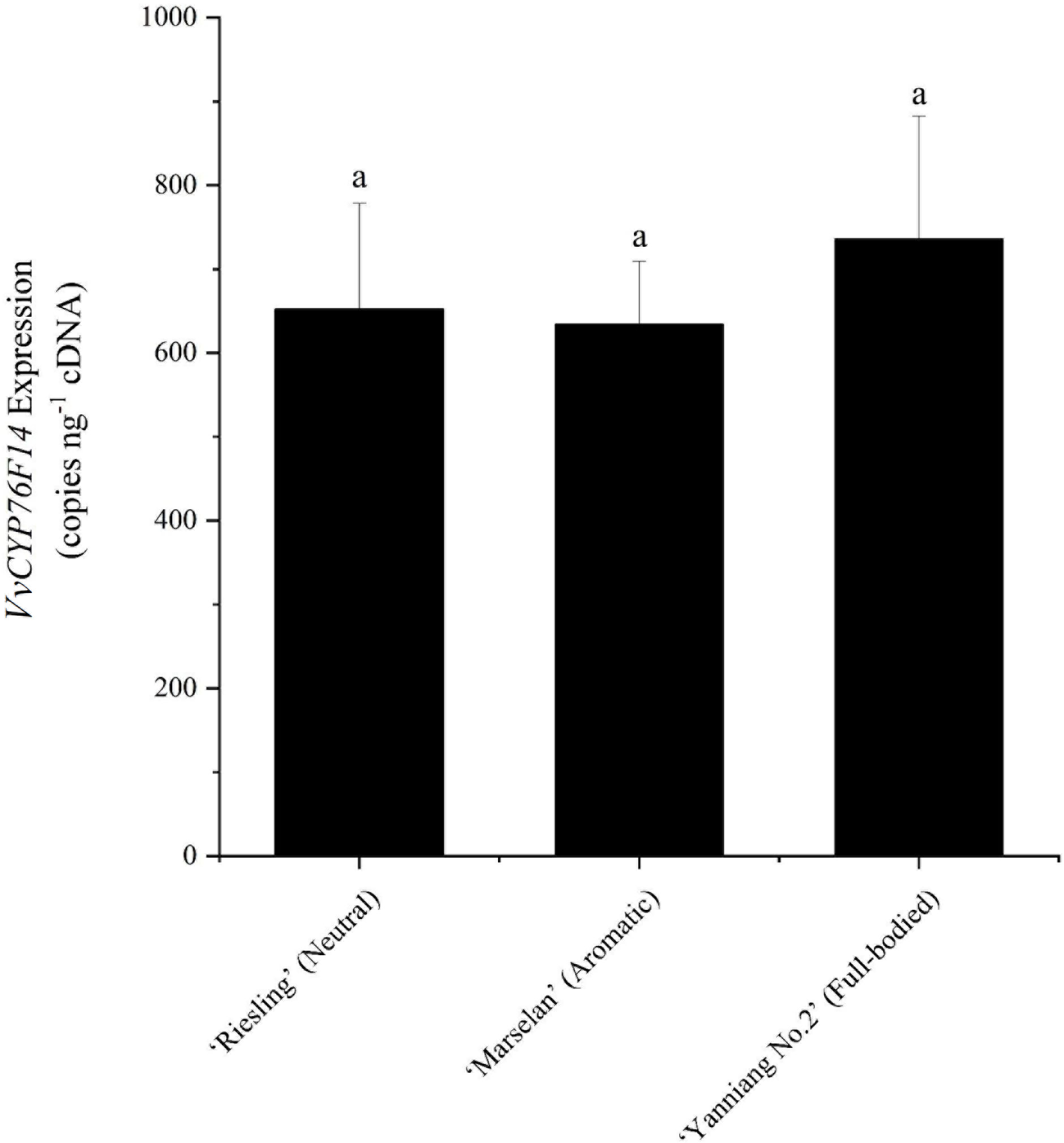

Supplement: Supplementary Figure 1 — Expression levels of VvCYP76F14s from three wine bouquet types of grape varieties. Data are presented as means ± SEs (n = 3). Letters represent significant differences at a significance level of p ≤ 0.05, as determined using ANOVA followed by Fisher’s LSD test. [file Image1.tif]
