# Supplementary material for: Cytochrome P450 VvCYP76F14 dominates the production of wine bouquet precursors in wine grapes
Source: Front Plant Sci. 2024 Oct 11;15:1450251. doi: 10.3389/fpls.2024.1450251 (PMC11502375; doi:10.3389/fpls.2024.1450251)
Supplement: Supplementary file 2 [file Table1.docx]

Supplementary Table 1. Different wine bouquet precursor contents among Neutral, Aromatic, and Full-bodied wine grape varieties.

| Variety | Italian Riesling | Marselan | Yanniang No.2 |
| --- | --- | --- | --- |
| Linalool | 5.92 ± 0.63 a | 5.84 ± 0.65 a | 6.02 ± 0.62 a |
| (*E*)-8-hydroxylinalool | 0.19 ± 0021 c | 1.38 ± 0.12 b | 3.76 ± 0.28 a |
| (*E*)-8-oxolinalool | 0.16 ± 0.024 c | 0.89 ± 0.092 b | 2.23 ± 0.21 a |
| (*E*)-8-carboxylinalool | 0.081 ± 0.0093 c | 0.34 ± 0.041 b | 0.92 ± 0.11 a |

The contents of linalool, (*E*)-8-hydroxylinalool, (*E*)-8-oxolinalool and (*E*)-8-carboxylinalool in the grape berries were determined using HPLC-HRMS. Data are presented as means ± SEs (*n* = 3). Letters represent significant differences among three wine bouquet type varieties at a significance level of *p* ≤ 0.05, as determined using ANOVA followed by Fisher’s LSD test.
