# Supplementary material for: Cytochrome P450 VvCYP76F14 dominates the production of wine bouquet precursors in wine grapes
Source: Front Plant Sci. 2024 Oct 11;15:1450251. doi: 10.3389/fpls.2024.1450251 (PMC11502375; doi:10.3389/fpls.2024.1450251)
Supplement: Supplementary file 3 [file Table2.docx]

Supplementary Table 2. The *in vitro* specific activity of the recombinant VvCYP76F14s from *E*. *coli*.

| Variety | Recombinant VvCYP76F14 of ‘Yanniang No.2’ | Recombinant VvCYP76F14 of ‘Italian Riesling’ | Recombinant VvCYP76F14 of ‘Marselan’ | Substrates without recombinant protein |
| --- | --- | --- | --- | --- |
| Linalool | 8.94 ± 1.37 c | 17.06 ± 1.98 b | 12.37 ± 1.82 c | 31.45 ± 1.87 a |
| (*E*)-8-hydroxylinalool | 7.33 ± 1.29 c | 14.28 ± 2.06 b | 12.32 ± 1.57 b | 29.38 ± 1.68 a |
| (*E*)-8-oxolinalool | 10.03 ± 1.84 b | 10.36 ± 1.99 b | 10.21 ± 2.01 b | 30.04 ± 1.42 a |

The *in vitro* specific activity of three wine bouquet type VvCYP76F14s was assessed by measuring the residual levels of substrates. Data are presented as means ± SE (*n* =3). Letters indicate significant differences among VvCYP76F14s from three wine bouquet type varieties at a significance level of *p* ≤ 0.05, as determined using ANOVA followed by Fisher’s LSD test.
