# Supplementary material for: Cytochrome P450 VvCYP76F14 dominates the production of wine bouquet precursors in wine grapes
Source: Front Plant Sci. 2024 Oct 11;15:1450251. doi: 10.3389/fpls.2024.1450251 (PMC11502375; doi:10.3389/fpls.2024.1450251)
Supplement: Supplementary file 4 [file Table3.docx]

Supplementary Table 3. Enzyme kinetics of VvCYP76F14 and its site-directed mutant proteins (VvCYP76F14-SMs) using linalool, (*E*)-8-hydroxylinalool and (*E*)-8-oxolinalool as substrate, respectively.

| Complexes | Enzyme | *k*_m_ (μM) | *k*_cat_ (S^-1^) | *k*_cat_/*k*_m_ |
| --- | --- | --- | --- | --- |
| VvCYP76F14-linalool | VvCYP76F14 | 66.24 ± 5.63 b | 13.12 ± 1.59 a | 0.22 ± 0.018 a |
|  | N46S | 66.11 ± 7.11 b | 13.65 ± 1.85 a | 0.21 ± 0.017 a |
|  | T107I | 65.12 ± 6.41 b | 12.18 ± 0.96 a | 0.19 ± 0.017 a |
|  | N111K | 69.31 ± 8.22 b | 12.55 ± 1.22 a | 0.18 ± 0.022 a |
|  | I120L | 151.25 ± 14.56 a | 7.71 ± 1.22 b | 0.051 ± 0.0037 b |
|  | R175Q | 69.86 ± 6.78 b | 10.76 ± 0.96 a | 0.18 ± 0.015 a |
|  | L222V | 67.52 ± 7.12 b | 13.51 ± 1.11 a | 0.20 ± 0.018 a |
|  | M264I | 67.58 ± 6.02 b | 12.25 ± 0.89 a | 0.18 ± 0.019 a |
|  | S286N | 71.25 ± 8.22 b | 12.22 ± 0.91 a | 0.17 ± 0.022 a |
|  | L298V | 67.58 ± 6.94 b | 0.094 ± 0.011 d | 0.0014 ± 0.00015 d |
|  | K325T | 67.26 ± 6.53 b | 11.15 ± 0.86 a | 0.17 ± 0.018 a |
|  | E378G | 144.32 ± 15.56 a | 1.32 ± 0.15 c | 0.0092 ± 0.0011 c |
|  | T380A | 160.21 ± 18.33 a | 6.86 ± 0.76 b | 0.043 ± 0.0051 b |
|  | E383D | 72.38 ± 6.91 b | 13.02 ± 1.11 a | 0.17 ± 0.022 a |
|  | T386A | 65.13 ± 7.26 b | 12.31 ± 1.34 a | 0.19 ± 0.021 a |
| VvCYP76F14-(*E*)-8-hydroxylinalool | VvCYP76F14 | 29.12 ± 2.66 b | 13.55 ± 1.16 a | 0.48 ± 0.039 a |
|  | N46S | 31.36 ± 1.14 b | 12.55 ± 0.85 a | 0.42 ± 0.062 a |
|  | T107I | 30.96 ± 1.62 b | 12.74 ± 1.41 a | 0.41 ± 0.051 a |
|  | N111K | 29.36 ± 2.81 b | 12.49 ± 1.00 a | 0.42 ± 0.053 a |
|  | I120L | 76.68 ± 7.22 a | 0.99 ± 0.12 bc | 0.013 ± 0.0018 bc |
|  | R175Q | 30.84 ± 1.08 b | 13.57 ± 1.42 a | 0.44 ± 0.039 a |
|  | L222V | 29.98 ± 2.71 b | 11.98 ± 1.20 a | 0.40 ± 0.053 a |
|  | M264I | 31.22 ± 3.77 b | 14.01 ± 1.11 a | 0.45 ± 0.051 a |
|  | S286N | 30.15 ± 2.88 b | 13.01 ± 1.00 a | 0.43 ± 0.053 a |
|  | L298V | 30.59 ± 2.85 b | 0.24 ± 0.031 c | 0.008 ± 0.00091 c |
|  | K325T | 30.73 ± 3.42 b | 12.58 ± 1.34 a | 0.41 ± 0.052 a |
|  | E378G | 65.01 ± 6.98 a | 0.97 ± 0.10 bc | 0.015 ± 0.0018 bc |
|  | T380A | 70.97 ± 7.92 a | 1.56 ± 0.17 b | 0.022 ± 0.0026 b |
|  | E383D | 29.87 ± 3.24 b | 12.84 ± 1.32 a | 0.43 ± 0.039 a |
|  | T386A | 31.95 ± 3.58 b | 12.45 ± 1.29 a | 0.39 ± 0.051a |
| VvCYP76F14-(*E*)-8-oxolinalool | VvCYP76F14 | 41.44 ± 3.93 b | 12.81 ± 1.42 a | 0.31 ± 0.029 a |
|  | N46S | 45.33 ± 5.10 b | 12.68 ± 1.37 a | 0.28 ± 0.035 a |
|  | T107I | 42.10 ± 4.14 b | 12.18 ± 1.26 a | 0.29 ± 0.033 a |
|  | N111K | 41.94 ± 3.03 b | 11.25 ± 1.66 a | 0.27 ± 0.034 a |
|  | I120L | 90.76 ± 12.24 a | 2.36 ± 0.31 b | 0.026 ± 0.0028 b |
|  | R175Q | 42.49 ± 3.44 b | 12.76 ± 1.32 a | 0.30 ± 0.035 a |
|  | L222V | 42.64 ± 5.08 b | 11.94 ± 1.28 a | 0.28 ± 0.041 a |
|  | M264I | 43.14 ± 4.56 b | 12.98 ± 1.51 a | 0.30 ± 0.032 a |
|  | S286N | 42.33 ± 4.52 b | 11.82 ± 1.23 a | 0.28 ± 0.037 a |
|  | L298V | 41.86 ± 5.24 b | 0.058 ± 0.0071 d | 0.014 ± 0.0017 bc |
|  | K325T | 41.88 ± 5.24 b | 12.09 ± 1.28 a | 0.29± 0.036 a |
|  | E378G | 88.52 ± 9.85 a | 0.85 ± 0.10 c | 0.0097 ± 0.0012 c |
|  | T380A | 94.79 ± 11.45 a | 1.89 ± 0.22 b | 0.020 ± 0.0023 b |
|  | E383D | 43.11 ± 4.22 b | 12.48 ± 1.39 a | 0.29 ± 0.031 a |
|  | T386A | 41.76 ± 3.05 b | 11.69 ± 1.25 a | 0.28 ± 0.043 a |

Data were presented as the means ± SE (*n* = 3). Letters represent significant differences among VvCYP76F14 and VvCYP76F14-SMs at a significance level of *p* ≤ 0.05, as determined using ANOVA followed by Fisher’s LSD test.
