# Supplementary material for: Cytochrome P450 VvCYP76F14 dominates the production of wine bouquet precursors in wine grapes
Source: Front Plant Sci. 2024 Oct 11;15:1450251. doi: 10.3389/fpls.2024.1450251 (PMC11502375; doi:10.3389/fpls.2024.1450251)
Supplement: Supplementary file 5 [file Table4.docx]

Supplementary Table 4. Transient expression of Full-Bodied type *VvCYP76F14* in three wine bouquet type varieties.

（A）

| ‘Yanniang No.2’ berries | Wild type | Empty vector | VvCYP76F14 |
| --- | --- | --- | --- |
| Linalool | 6.01 ± 0.62 a | 5.83 ± 0.67 a | 3.52 ± 0.41 b |
| (*E*)-8-hydroxylinalool | 3.76 ± 0.25 b | 3.62 ± 0.35 b | 4.91 ± 0.44 a |
| (*E*)-8-oxolinalool | 2.23 ± 0.21 b | 2.12 ± 0.26 b | 2.89 ± 0.27 a |
| (*E*)-8-carbooxylinalool | 0.92 ± 0.11 b | 0.88 ± 0.093 b | 1.26 ± 0.13 a |

（B）

| ‘Marselan’ berries | Wild type | Empty vector | VvCYP76F14 |
| --- | --- | --- | --- |
| Linalool | 5.84 ± 0.65 a | 5.91 ± 0.55 a | 3.45 ± 0.42 b |
| (*E*)-8-hydroxylinalool | 1.38 ± 0.12 b | 1.41 ± 0.15 b | 1.98 ± 0.24 a |
| (*E*)-8-oxolinalool | 0.89 ± 0.096 b | 0.92 ± 0.11 b | 1.29 ± 0.13 a |
| (*E*)-8-carbooxylinalool | 0.34 ± 0.041 b | 0.36 ± 0.043 b | 0.51 ± 0.052 a |

（C）

| ‘Italian Riesling’ berries | Wild type | Empty vector | VvCYP76F14 |
| --- | --- | --- | --- |
| Linalool | 5.92 ± 0.63 a | 5.75 ± 0.61 a | 2.86 ± 0.32 b |
| (*E*)-8-hydroxylinalool | 0.19 ± 0.021 b | 0.21 ± 0.023 b | 0.31 ± 0.034 a |
| (*E*)-8-oxolinalool | 0.16 ± 0.024 b | 0.17 ± 0.025 b | 0.22 ± 0.027 a |
| (*E*)-8-carbooxylinalool | 0.081 ± 0.0093 b | 0.085 ± 0.0091 b | 0.11 ± 0.013 a |

Data are presented as means ± SEs (*n* = 3). Letters represent significant differences among wild type berries, berries transformed with empty vector, and berries transformed with *VvCYP76F14* at a significance level of *p* ≤ 0.05, as determined using ANOVA followed by Fisher’s LSD test.
